# Revisiting the membrane-centric view of diabetes

**DOI:** 10.1186/s12944-016-0342-0

**Published:** 2016-09-27

**Authors:** Marc Pilon

**Affiliations:** Department of Chemistry and Molecular Biology, University of Gothenburg, Medicinaregatan 9, Box 462, S-405 30 Göteborg, Sweden

**Keywords:** PAQR-2, Membrane fluidity, Diabetes, AdipoR1, AdipoR2, Phospholipid, Fatty acids, Diet, Lipidomics

## Abstract

Fundamental questions remain unresolved in diabetes: What is the actual mechanism of glucose toxicity? Why is there insulin resistance in type 2 diabetes? Why do diets rich in sugars or saturated fatty acids increase the risk of developing diabetes? Studying the *C. elegans* homologs of the anti-diabetic adiponectin receptors (AdipoR1 and AdipoR2) has led us to exciting new discoveries and to revisit what may be termed “*The Membrane Theory of Diabetes”*. We hypothesize that excess saturated fatty acids (obtained through a diet rich in saturated fats or through conversion of sugars into saturated fats via lipogenesis) leads to rigid cellular membranes that in turn impair insulin signalling, glucose uptake and blood circulation, thus creating a vicious cycle that contributes to the development of overt type 2 diabetes. This hypothesis is supported by our own studies in *C. elegans* and by a wealth of literature concerning membrane composition in diabetics. The purpose of this review is to survey this literature in the light of the new results, and to provide an admittedly membrane-centric view of diabetes.

## Background

### Diabetes and new insights from *C. elegans*

The worldwide rise in the incidence of type 2 diabetes is a recent phenomenon that coincides with lifestyle changes during the 20th century. A diet of excess combined with an increasingly sedentary lifestyle clearly leads to an energy imbalance and the accumulation of fat depots. While there is no doubt that obesity and genetic variants are risk factors for developing type 2 diabetes, what, precisely, is the molecular and cell biology link between diet and diabetes? Many explanations have been proposed. Here, we revisit a “membrane-centric” view of diabetes because of some new results obtained with the small nematode worm *C. elegans*. Specifically, as little as 10 mM glucose is lethal to *C. elegans* mutants lacking a functional homolog of the mammalian adiponectin receptors [1–3]. This toxicity is accompanied by an increase in the abundance of saturated fatty acids (SFAs) in membrane phospholipids and a dramatic decrease in membrane fluidity. Given the proposed anti-diabetic activities of the adiponectin receptors [4–8], the *C. elegans* studies prompted us to examine the literature for possible connections between glucose toxicity, cellular membranes and diabetes.

## Decreased membrane fluidity in diabetics

Red blood cells (RBCs) in diabetics are abnormally rigid. This fact is known since at least 1978 when purified RBCs were filmed as they deformed under different, quantifiable amounts of air pressure inside glass microcapillaries [9]. These findings were confirmed independently using a filtration rate assay [10], and more recently using high-speed filming of RBCs through microchannels [11]. The decreased deformability of RBCs is a likely source of shear stress that contributes to microcapillary hardening in diabetics, an idea proposed in 1978 by McMillan et al. [9]. Several methods were later used to show that the low deformability of RBCs in diabetics is caused by a reduced fluidity of the cellular membranes. Already in 1979, Baba et al. measured depolarization of a fluorescent probe and found reduced membrane fluidity in the RBCs of diabetics [12]. Similar findings were made in 1983 by Kamada and Otsuji, this time using spin labeling and electron spin resonance measurements [13]. Kamada et al. also showed that newly produced RBCs in diabetics start off with an already reduced fluidity, indicating that the low fluidity is not a result of faster decay of the RBCs in diabetics, but rather likely reflects a basic problem with the pool of fatty acids (FAs) available for membrane homeostasis [14]. Most studies of membrane properties are done on RBCs because of their easy availability. However, decreased membrane fluidity in diabetics has also been measured in several other cell types, including ileal enterocytes of the intestinal brush border [15, 16], the sarcolema of cardiac myocytes [17], leukocytes [18], synaptic vesicles in the cerebral cortex [19] and platelets [20, 21], and is likely affecting most cell types.

## Abnormal phospholipid composition in diabetics

Phospholipid composition has a great influence on membrane properties. An excellent proof of this is homeoviscous adaptation in poikilotherms or deep water organisms: temperature [22, 23] and hydrostatic pressure [24–26] can have profound effects on membrane fluidity to which cells adapt by compensatory changes in lipid composition, a phenomenon termed “homeoviscous adaptation” [22, 23, 27]. Specifically, certain lipid types increase membrane fluidity (e.g., phospholipids containing unsaturated fatty acids (UFAs)), while others decrease it (e.g., cholesterol, ceramides and phospholipids containing saturated fatty acids (SFAs)) [28–33].

Several independent studies have found that the cellular membranes of diabetics are rich in rigidity-promoting lipids: excess cholesterol [34], excess sphingomyelin [35], and excess SFAs [36–39] have all been associated with diabetes. Even more tantalizing is the predictive power of lipid composition. At least two large longitudinal studies measured the phospholipid composition of RBCs in thousands of healthy subjects and followed them for several years [40–42]. In both studies, individuals with the highest proportion of SFAs were most likely to later develop type 2 diabetes. This suggests that low membrane fluidity may precede diabetes. However, like many other observations mentioned in this review, it remains to be seen to what degree this constitutes a “marker” or a “maker” of imminent diabetes.

## Low membrane fluidity as a cause of diabetes

Optimal membrane properties are essential for numerous cellular processes that are often defective in diabetics: vesicular trafficking (including insulin secretion by beta cells [43]), glucose transport [44], endocytosis [45, 46], regulation of metabolic rate [47], platelet aggregation [20], etc. Of special interest in the context of diabetes is the importance of membrane fluidity on the function of membrane proteins. Several studies have shown that insulin receptor signaling is impaired by low membrane fluidity, probably because lateral diffusion and localization to membrane microdomains is important for ligand binding and signaling [44, 48–50]. This is particularly important for two reasons: 1) it has long been known that insulin signaling activates FA desaturases in the liver and is thus important for regulating membrane fluidity [51–54]; and 2) insulin signaling is important for inducing the transport of GLUT4 to the plasma membranes of muscle cells and adipocytes, which is essential for quick clearance of blood glucose [55]. Also important is that GLUT4 transport to the plasma membrane is itself impaired by decreased membrane fluidity, which further exacerbates the problems of glucose clearance [50, 56]. Defects in phospholipid membrane composition of adipocytes is also responsible for inflammation and limits the insulin-induced expansion of adipose tissues in obese human [57]. Thus, a state of low membrane fluidity is very much diabetes-prone since it impairs insulin signaling and response.

## Effect of diet on membrane composition

Several studies have shown that the composition of membrane phospholipids is influenced by the fatty acid composition of the diet. For example, fish oil supplements lead to an increased abundance of polyunsaturated fatty acids (PUFAs) in RBC membranes [58, 59]. Also, rats fed with diets differing in their FA composition (i.e., a range of SFA: UFA ratios) show phospholipid compositions that reflects the dietary fats [60, 61]. Similar findings were made with human subjects assigned to diets differing in their FA composition [62]. These results show that dietary fatty acids can be directly incorporated into phospholipids. Consequently, a diet rich in SFAs will tend to reduce the fluidity of cellular membranes. This is also true of a carbohydrate-rich diet since glucose can readily be converted into SFAs via de novo lipogenesis (DNL) in liver and adipocytes, which can then be made available throughout the body via the bloodstream as lipids transported in lipoproteins, or as free fatty acids [63]. The so-called “Western diet” therefore may promote diabetes by lowering membrane fluidity, hence impairing insulin signaling and other processes. Conversely, improvements in membrane fluidity may explain the insulin-sensitizing benefits of PUFA-rich diets [64, 65].

Incidentally, DNL is tightly associated with desaturation of the newly synthesized SFAs so as to create a balanced composition of the FA pool. This is evidenced from the observation that supplementing cultivated adipocytes with the SFA palmitate activates a membrane-protective desaturase activity that is paradoxically accompanied by the coordinated activation of the entire DNL pathway, which produces even more palmitate [66]. However, while the enzymes for DNL are restricted to a few tissues, most cells express one or two desaturases [67–69]. Why? Probably so that each cell can locally adjust its mix of SFAs, monounsaturated fatty acids (MUFAs) and PUFAs available for membrane turnover, as we shall now discuss.

## Regulation of membrane fluidity

Regulatory mechanisms must exist within each cell to adjust membrane composition and maintain near-optimal properties. This is evident from the fact that substantial changes in dietary fatty acid composition are usually required for relatively small changes in membrane composition. To put it bluntly: without such regulatory mechanisms, some of us would be butter-like solids at room temperature while other would be oil-like liquids, depending on whether our diets are rich in animal fats or vegetable oils. In spite of their obvious importance, it is only in recent years that molecular regulators of membrane composition have been identified. First came the discovery of the bacterial fluidity regulator DesK, a kinase that activates a fatty acid desaturase upon reduced membrane fluidity; this helps restore membrane fluidity during homeoviscous adaptation to low temperature [70–75]. In the yeast *Saccharomyces cerevisiae,* the transmembrane protein Mga2 was found to act as a sensor for endoplasmic reticulum (ER) membrane lipid saturation: it is cleaved when ER membranes become too rigid, thus releasing a transcription factor domain that activates expression of a Δ9 fatty acid desaturase, hence restoring membrane fluidity [76]. Finally, a plasma membrane fluidity regulator was recently identified in the nematode *C. elegans* and consists of at least two proteins, PAQR-2 and IGLR-2 that are homologs of the ubiquitously expressed human adiponectin receptors and LRIG-type proteins, respectively [1–3, 77]. The mechanism of fluidity sensing is not known for PAQR-2/IGLR-2. However, our work in *C. elegans* suggests that PAQR-2 improves membrane fluidity by causing the upregulation of FA desaturases, likely via ligand-regulated transcription factors such as NHR-49 (a functional ortholog of the mammalian PPARα) and SBP-1 (an ortholog of the mammalian SREBP). Based on sequence homology and structural considerations, we and others suspect that PAQR-2 and the mammalian adiponectin receptors are hydrolases acting on a lipid substrate to release a ligand that regulates downstream transcription factors [2, 77–79]. An alternative explanation, inspired by studies on a yeast homolog, is that the adiponectin receptors act as ceramidases that deplete fluidity-lowering ceramides and release the signaling molecule sphingosine-1-phosphate [6, 80].

Interestingly, worms lacking PAQR-2 or IGLR-2 are the most glucose-intolerant *C. elegans* mutants identified so far: they succumb in the presence of as little as 10 mM glucose [3]. In these mutants, glucose causes a dramatic accumulation of SFAs in membranes with a concomitant loss of membrane fluidity, and this is likely due to the conversion of glucose into SFAs via DNL. This shows that the PAQR-2/IGLR-2 complex is essential for homeoviscous adaptation in the presence of glucose, with obvious implications for diabetes. Many membrane-related phenotypes of the PAQR-2 or IGLR-2 deficient worms, including cold and glucose intolerance, can be suppressed by the inclusion of small amounts of non-ionic detergents, such as NP-40 or Triton X-100, which act as membrane fluidizers. This is especially interesting because metformin, an antihyperglycemic agent already commonly used to treat diabetes, may also act by improving membrane fluidity [81–84]. It will therefore be very interesting to define the roles of the mammalian homologs of PAQR-2/IGLR-2 in the context of the carbohydrate and SFA-rich Western diet, and diabetes. Specifically: is the mammalian homolog of the PAQR-2/IGLR-2 complex constantly “playing catch-up” to compensate for the fluidity-lowering effects of the Western diet? Could the well-documented diabetes-preventing effects of adiponectin and its receptors be explained by their roles in membrane homeostasis?

## Genetics

Like all human traits, propensity to develop type 2 diabetes is influenced by genetic variation [85]. Several loci likely to have an impact on membrane fluidity have been linked to type 2 diabetes. In particular, several studies have linked polymorphisms in desaturase activity to abnormal fatty acid composition and type 2 diabetes risk [86–88]. Several polymorphisms in adiponectin or its receptors have also been linked to insulin resistance [89–93]. One study of special interest established a provocative correlation between certain single-nucleotide polymorphorphisms in adiponectin and AdipoR1 with the levels of plasma SFAs and insulin resistance [89]. The authors of this study concluded that “Personalized dietary advice to decrease SFA consumption in these individuals may be recommended as a possible therapeutic measure to improve insulin sensitivity.” There is also genetic evidence for the “flip side” of the membrane fluidity coin: Greenland Inuits with a highly fluidity-promoting omega-3 fat-rich diet show strong signs of positive selection for reduced-activity variants of delta-5 and delta-6 desaturases [94], which essentially reduces their ability to generate excessively fluid membranes.

## Conclusions

Here then is a bite-sized theory that attempts to weave together the observations listed above into a “*Membrane Theory of Diabetes*”. SFAs obtained from the diet or via lipogenesis in the liver and adipocytes pose a relentless challenge to fluidity-sustaining systems, even more so in genetically predisposed individuals. This is likely exacerbated by unnatural fats of various types generated during the production of margarines or superheated vegetable oils used for frying much of our (fast) foods, and which may not be handled efficiently by the cellular machinery [95–97]. Chronic low fluidity in our membranes has several diabetes-promoting consequences, including impairing insulin secretion and signaling, reduced efficacy of GLUT4 localization to membranes and hardening of blood vessels. The idea that low membrane fluidity is an important component of diabetes pathophysiology is an old one that has been reviewed a few times [50, 56, 88, 98–100]. However, the recent identification of eukaryotic regulators of membrane fluidity should revive interest in this subject since they open novel experimental and therapeutic avenues.

## Abbreviations

DNL: De novo lipogenesis
ER: Endoplasmic reticulum
FAs: Fatty acids
FRAP: Fluorescence recovery after photobleaching
MUFAs: Monounsaturated fatty acids
PUFAs: Polyunsaturated fatty acids
RBCs: Red blood cells
SFAs: Saturated fatty acids
UFAs: Unsaturated fatty acids
